# The effect of disinfectant ingredients on teat skin bacteria associated with mastitis in Irish dairy herds

**DOI:** 10.1186/s13620-020-00179-7

**Published:** 2021-01-02

**Authors:** Sarah Rose Fitzpatrick, Mary Garvey, Jim Flynn, Bernadette O’Brien, David Gleeson

**Affiliations:** 1 Teagasc, Animal & Grassland Research and Innovation Centre, Moorepark, Fermoy, County Cork Ireland; 2 Department of Life Science, Institute of Technology Sligo, County Sligo, Ireland

**Keywords:** Teat disinfection, mastitis, teat swabbing, bacterial load

## Abstract

**Background:**

Teat disinfection is an important step in the control of mastitis within a dairy herd. The objective of this study was to evaluate the effectiveness of 96 commercially available teat disinfectant products in Ireland against bacterial isolates on teat skin. Teat disinfection products were applied to the teats of seventeen Holstein–Friesian cows. A split-udder model was used where one cow received two different teat disinfection products on each day. A composite swab sample was taken of the left teats and the right teats before and after teat disinfectant application. Swab samples were plated onto 3 different selective agars to enumerate bacterial counts of streptococcal, staphylococcal and coliform isolates.

**Results:**

Streptococcal isolates were the most prominent bacterial group recovered on teat swabs taken before the application of a teat disinfection product (55.0%), followed by staphylococcal isolates (41.3%) and coliform isolates (3.7%). Products were reclassified by active ingredients (n = 9) for analysis. These ingredient groups included; chlorhexidine, chlorine dioxide, diamine, iodine, iodine and lactic acid, lactic acid, lactic acid and chlorhexidine, lactic acid and hydrogen peroxide, and lactic acid and salicylic acid. The ingredient group, chlorine dioxide, resulted in comparable reductions to the iodine group for streptococcal isolates. The ingredient group, iodine combined with lactic acid, resulted in the greatest reduction of staphylococcal isolates. When observing products individually, a product containing 1.6% w/w lactic acid combined with hydrogen peroxide was the most effective at reducing streptococcal isolates on the teat skin, whereas a product containing lactic acid combined with 0.6% w/w chlorhexidine was the most effective against staphylococcal isolates. Minor differences were observed regarding the relationship between effectiveness and active ingredient concentration between products.

**Conclusions:**

This study suggests that some teat disinfectant products achieve a higher reduction in bacterial levels against different specific bacterial groups on teat skin than other products. Therefore, when choosing a teat disinfectant product, the bacteria in the dairy herds’ environment should be considered. Further studies are necessary to evaluate products efficacy against new IMIs and any possible effects on teat skin condition.

## Introduction

Mastitis is one of the main milk production and economic problems facing the global industry [1] due to reduced milk production, the need to discard abnormal milk or treated milk containing antibiotics, premature culling, veterinary services and the treatment of mastitis. Using a 70 cow herd as an example, the CostCheck calculator showed a reduction in net farm profits of approximately €10,688 associated with an average herd SCC of 200,000-300,000 cells/mL compared to a target of 100,000–200,000 cells/mL [2]. Mastitis can also have an adverse effect on milk quality, animal health and welfare [3], an important issue to address to satisfy the One Health approach. Many bacterial strains have been associated with mastitis, with the main strains being identified as *Streptococcus agalactiae*, *Streptococcus dysgalactiae* and *Streptococcus uberis*, *Staphylococcus aureus*, and *Escherichia coli* [4]. In Ireland, the bacteria associated with mastitis are *Staphylococcus aureus*, *Streptococcus uberis* and *Escherichia coli* [5]. Teat disinfection reduces infection rates with causative agents by reducing the bacterial load on the teat skin surface [6, 7] and it has become a central part of the milking routine of the modern herd [8].

Teat skin of cows can represent the source of bacterial populations found in raw milk [9], with the rate of mastitis and intramammary infections (IMIs) having previously been shown to increase with increasing bacterial numbers on the teat skin [6, 10]. Pre- and post-milking teat disinfectants perform differently in reducing bacterial transfer from the cow’s environment (i.e. bedding, housing, yards) or between cows. Pre-milking teat disinfection has been shown to be effective at reducing the incidence of mastitis caused by environmental bacteria i.e. *Str*. *uberis* and *E*. *coli* [11, 12] and may decrease the cow infection ratio by reducing udder bacterial contamination from the environment [13]. Alternatively, post-milking teat disinfection has been shown to reduce the IMIs caused by contagious bacteria, such as *Staphylococcus aureus* [14] which may have been transferred during milking via the milkers [15] or the milking machine [16]; post-milking disinfection has proven less effective against environmental bacteria, such as coliforms and some streptococci species [17].

Before teat disinfectant products can be sold commercially in Ireland, the product must be registered with the Department of Agriculture, Food and Marine (DAFM) and with the Health Products Regulatory Authority (HPRA), and must also comply with European legislation. According to HPRA, if there are no medical claims made, the disinfectant product intended for the application to skin for general hygiene may be classified as a biocide rather than as a veterinary medicine [18] and so subject to registration under Biocidal Products Regulation (Regulation (EU) No 528 of 2012). Within the European Union, member states must use a common standard to evaluate teat disinfectant products.

This European standard (EN) which is known as the BS EN 1656 can be used to compare a range of disinfectants. However, there are also other laboratory and field methods by which teat disinfectant products can be screened. Previous studies have used modified versions of the BS EN 1656 [19] and the disc diffusion method [20, 21] to screen and evaluate teat disinfectant products in short-term laboratory tests. However, laboratory methods cannot evaluate the impact of the teat disinfectant product on the bacterial load on the teat skin or efficacy in reducing new IMIs. Alternatively, field tests can evaluate the effect (positive or negative) of a teat disinfectant product on the reduction of new IMIs or the bacterial load on the teat skin; field test methods include natural exposure and teat swabbing. The natural exposure protocol is suitable for application on commercial herds and can be used to evaluate the efficacy of a teat disinfectant product in reducing the incidence of new IMI’s over a period of 12 weeks and over a full lactation [22–24]. However, the practicality of this method could be limited for testing a large number of teat disinfectants within a certain time period. Teat swabbing has been previously used to determine the effectiveness of a pre-milking cleaning regime [25, 26] and also to determine the effectiveness of teat disinfectant products in reducing the bacterial load on teat skin surfaces [27]. This method allows for the testing of many products over a short time frame.

The majority of research studies in the past have been undertaken in relation to iodine-based teat disinfectants [28, 29]. However, the use of iodine-based products can result in high iodine concentrations in milk, which may concern infant formula manufacturers [30]. Furthermore, there are many new commercially available teat disinfectant products in Ireland. These products contain a range of active ingredients including iodine, chlorhexidine, lactic acid, chlorine dioxide and salicylic acid, with various combinations of these ingredients. The objective of the present study was to evaluate the effectiveness of 96 commercially available teat disinfectant products of varying ingredients and concentrations in reducing mastitis-causing bacteria on teat skin using a teat swab method.

## Materials and Methods

### Teat Disinfectant Information

Ninety-six commercially available teat disinfectant products, with different active ingredients of varying concentrations, were tested against bacteria, isolated from the teat skin of Irish dairy cattle, using a teat skin swabbing method. The teat disinfectant products were either ready-to-use (RTU) (*n* = 82) concentrate (conc.) products (*n* = 9) or required activation before use (*n* = 5). Concentrate products were diluted to a usable concentration, using sterile distilled water (according to the manufacturer’s recommendation), to avoid possible issues with water hardness or contaminated water. Five chlorine dioxide products (Product number 11, 70, 89, 90 and 95) were mixed with an activator before use, according to the manufacturer’s recommendations. The disinfectant products used were recommended either for both pre- and post-milking teat disinfection (*n* = 49), pre-milking teat disinfection only (*n* = 3), or post-milking disinfection only (*n* = 44). These products were all tested under the same environmental conditions i.e. the same milking parlour, cows, farm, climate conditions and with the same laboratory methods to ensure comparable results regarding the effectiveness of the different teat disinfectant products. Information regarding the products used is shown in Table 1.

### Test Methods

Ninety-five different teat disinfectant products were applied to the teats of spring calving Holstein-Friesian cows from the Teagasc Moorepark research farm, County Cork, Ireland in November 2019. This study was approved by the Teagasc Animal Ethics Committee (ref. TAEC168-2017). Seventeen cows, which were free from clinical mastitis infection, were chosen for the application of the test teat disinfectant products over a period of nine days. These cows were housed indoors, on matted cubicle beds dressed with ground limestone daily. A split udder design was used where each cow received two different teat disinfectant products at each sampling point before morning milking (one product applied to the two left teats and a different product applied to the two right teats). Each teat disinfectant was applied for three test days during the trial to three different cows.

Before sampling, teats were left unprepared and swabs (Copan Italia S.p.A Via F. Perotti, 10 25,125 Bresica – Italy) were moistened in sterile trypticase soy broth (TSB) (Merck Millipore, Ireland) to aid in the recovery of bacteria from the teat skin. A swab sample was then taken to enumerate the number of bacteria on the unprepared teat skin (PRE). A separate swab was used to collect a composite sample from the left teats (left front [LF] and left hind [LH]) while a separate swab was used to collect a composite swab sample from the right teats (right front [RF] and right hind [RH]). Teat disinfectant products were applied using non-return teat dip cups and the teats were immersed in the product. One product was applied to the 2 left teats of the cow and the other product applied to the remaining 2 teats on the right side. To standardise the evaluation of all product types, teats were wiped with an individual disposable paper towel, after approximately 1 min of disinfectant contact time. Two swabs were then used (one swab for LF and LH teats and one swab for RF and RH teats) to obtain a count of the bacteria left on the teats (POST).

The wiping of teats with an individual paper towel one minute after the application of disinfectant and before post swab samples was required to remove excess teat disinfectant from the teat skin. To establish if the application of a wet substance followed by drying with paper towels had any impact on the bacterial levels recovered, sterile phosphate-buffered saline (PBS) was applied onto the teat skin using a sterile dip cup; after one minute the teats were dried using individual paper towels. Sampling was carried out in the same manner as the teat disinfectants above. This was carried out prior to the commencement of the current study to determine the possible reduction of bacterial load due to the presence of a liquid solution and wiping action.

Teat swabbing involved drawing the swabs across the teat orifice and down the side of each teat avoiding contact with the udder hair or cows flank at all times. Teat swabbing was carried out by the same two operators for all treatments. In all, sampling provided 576 individual teat swab samples (96 products x 2 swabs/day x 3 test days), giving a total of 3 before and 3 after swab samples per treatment. Immediately after sampling, swabs were placed into individual sterile bottles containing 10 mL of sterile TSB and neutraliser (30 g/L polysorbate 80 & 3 g/L l-a-phosphatidylcholine from egg yolk). The TSB and neutraliser were prepared in 250 mL lots and autoclaved at 121 ˚C for 15 min, and then distributed into 10 mL aliquots in a laminar flow cabinet. The sterile bottles (containing TSB, neutraliser and swab) were placed in storage at -20 ˚C until analysed for the presence of staphylococcal and streptococcal isolates.

Before the start of analysis, the sterile bottles were defrosted and vigorously shaken on a vortex. To measure bacterial levels, dilutions of 1:100 were performed for the PRE samples using maximum recovery diluent in sterile tubes. The POST samples were used undiluted. The samples were subsequently plated, in triplicate, onto three separate agars; Baird parker agar (Merck KGaA64271, Darmstadt, Germany) with the addition of egg yolk tellurite emulsion for staphylococcal isolates, modified Edwards agar (Oxoid 3M0027) with 5% sterile sheep blood for streptococcal isolates and MacConkey agar (Merck Millipore, Ireland) for coliform isolates. Specific bacteria types within each category were not defined. Following incubation at 37 ˚C for 24 hrs, microbial counts for each isolate type were manually counted.

### Statistical Analysis

Statistical analysis was carried out using SAS for Windows, version 9.4. Bacterial counts (cfu/mL) were transformed to base-10 logarithm for analysis. Teat disinfectant effectiveness was defined as the Log_10_ percent reduction from the PRE swab for each teat disinfectant product using the following equation$\frac{100\left({Log}_{10}PRE swab- {Log}_{10}reduction \right)}{{Log}_{10}PRE swab}$ [28, 31]. Log_10_ reduction refers to the difference between the Log_10_ values of PRE and POST swabs. The application of sterile PBS and teat wiping resulted in a reduction of 0.8% for streptococcal isolates and 0.3% for staphylococcal isolates. These values were subtracted, according to isolate group, from the results obtained for each individual teat disinfectant product to account to the effect of the liquid applied and wiping action used in the study. PROC GLIMMIX was used to perform multiple pair-wise comparisons. The LSMEANS statement in PROC GLIMMIX was used to differentiate statistical differences. Products used within the study were categorised into groups based on the active ingredient (*n* = 9) to minimise/control the occurrence of Type II errors during analysis. These groups included; chlorhexidine (*n* = 25), chlorine dioxide (*n* = 5), diamine (*n* = 1), iodine (*n* = 13), iodine combined with lactic acid (*n* = 5), lactic acid (*n* = 15), lactic acid combined with chlorhexidine (*n* = 21), lactic acid combined with hydrogen peroxide (*n* = 1), and lactic acid combined with salicylic acid (*n* = 10). Products within the active ingredient group were compared to each other. The reductions for each bacterial isolate tested were analysed separately using the same model. This model included the Log_10_ percentage reductions as a dependant variable, and product within the active ingredient group and day as independent variables. The equation for the model was as follows; *Reduction = Product + Bacteria + Day + Product* X *Bacteria*, where reduction was the Log_10_ percentage reduction, where product was the teat disinfectant product tested and day was the date of sampling. The cow was the experimental unit. Differences between ingredient groups were analysed using the following model; *Reduction = Ingredient + Bacteria + Day + Ingredient* X *Bacteria*. Residual checks were made to ensure assumptions of analysis were met.

## Results

**Fig. 1:**
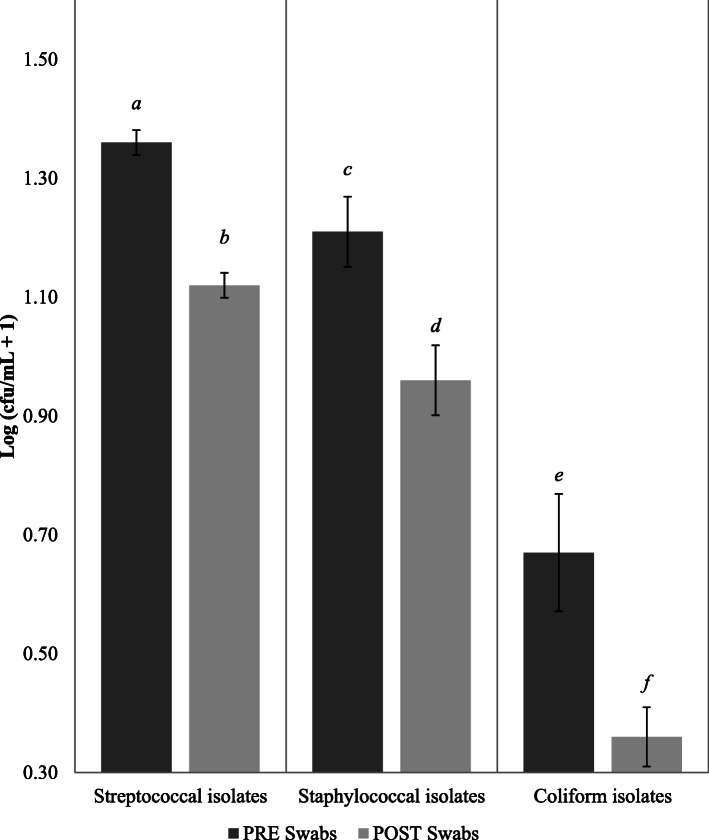
Overall Log_10_ bacterial counts for streptococcal, staphylococcal and coliform isolates on teat swabs before (PRE) and after (POST) the application of teat disinfection products. ^*a-f*^ Means with different letters differ significantly. Error bars indicate SEM

Streptococcal isolates were the most prominent bacterial group recovered on PRE teat swabs (55.0%), followed by staphylococcal isolates (41.3%) and coliform isolates (3.7%) (Fig. 1). The level of coliform isolates recovered was low and therefore not included in further analysis within this study. All teat disinfectant products tested had a positive effect on reducing the bacterial load on the teat skin. For streptococcal isolates, average Log_10_ bacterial reduction on teat skin was 36.6%. For staphylococcal isolates, average Log_10_ bacterial reduction on teat skin was 44.1%. Additionally, a sampling day and a day by product effect was observed within the data which shows there was a day to day variation of the level of bacterial contamination on teat skin.

**Fig. 2:**
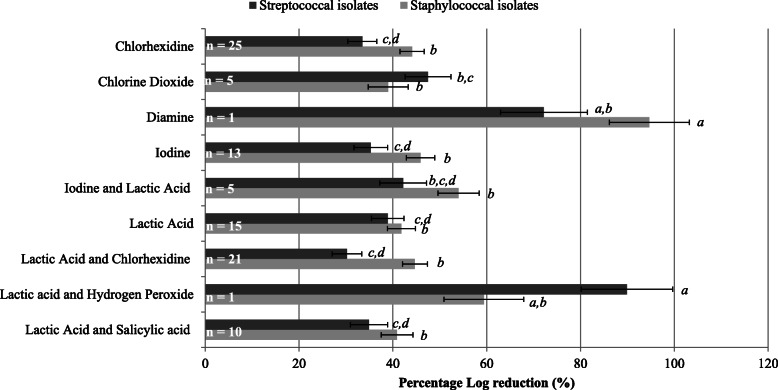
LS-means of the percentage Log_10_ reduction (%) of streptococcal and staphylococcal isolates across all active ingredients tested on the teat skin. Error bars indicates SEM. Percentage log reduction (%) determined from Log_10_ PRE swab values.^*a-d*^Means with different letters differ significantly

Within the study, there were nine ingredient groups created based on active ingredient: chlorhexidine, chlorine dioxide, diamine, iodine, iodine combined with lactic acid, lactic acid, lactic acid combined with chlorhexidine, lactic acid combined with hydrogen peroxide, and lactic acid combined with salicylic acid. Bacterial isolate LS-means percentage log reductions achieved by products within ingredient groups are summarised in Fig. 2. For two ingredient groups analysed, there was one only one product analysed per group. These ingredient groups included diamine and lactic acid combined with hydrogen peroxide. While it is recognised that these two groups had a limited amount of products, these groups achieved some of the greatest reductions in streptococcal and staphylococcal isolates. For streptococcal isolates, the ingredient group that contained a combination of lactic acid and hydrogen peroxide resulted in the largest bacterial reductions (89.9%). Lactic acid combined with chlorhexidine group resulted in the smallest bacterial reduction of 30.2%. For staphylococcal isolates, the ingredient group diamine resulted in a reduction of 94.7%, whereas, chlorine dioxide resulted in the smallest reduction of staphylococcal isolates (39.0%). The LS-means Log_10_ percentage (%) reduction for streptococcal and staphylococcal isolates for each individual product can be observed in Table 1.

For both streptococcal and staphylococcal isolates, no significant difference was observed between chlorine dioxide, iodine, chlorhexidine or lactic acid only. For streptococcal isolates, chlorine dioxide (47.5%) had numerically higher bacterial reductions than chlorhexidine (33.5%) and iodine (35.3%). Additionally, lactic acid combined with chlorhexidine resulted in the lowest overall reduction (30.2%) for streptococcal isolates. A combination of iodine and lactic acid was found to be the most effective (54.0%) against staphylococcal isolates, with chlorine dioxide resulting in a lower reduction (39.0%) which was not significantly different (P > 0.05). Despite products not being compared individually, it is possible to observe the most effective teat disinfectant product against both streptococcal and staphylococcal isolates. For streptococcal isolates, product 46 (1.6% w/w lactic acid and hydrogen peroxide), product 23 (2% w/w lactic acid and 0.1% w/w salicylic acid) and product 83 (0.5% w/w iodine) resulted in the highest percentage log reductions of 89.9%, 77.6% and 73.7%, respectively. For staphylococcal isolates, products containing 2% lactic acid combined with 0.6% w/w chlorhexidine (product 47), 0.6% w/w diamine (product 26) and chlorhexidine only (product 61) resulted in the highest percentage log reductions of 100%, 94.7% and 82.5%, respectively.

**Table 1:** LS-means of Log_10_ percentage reduction (%) for each teat disinfection product, within each ingredient group, against streptococcal and staphylococcal isolates

Product	#	Ingredient (w/w) ^1^	Pre or Post	*Strep* Isolates	*Staph* isolates
**Chlorhexidine Products**					
**Arrabawn Udder Guard**^**^**^	40	0.5% Chlorhexidine	Pre & Post	65.7 ^*a*^	56.8 ^*a,b*^
**C-Dip**^**^**^	61	0.53% Chlorhexidine	Post	19.8 ^*a,b*^	82.5 ^*a*^
**Deosan Mastocide**^**^**^	32	0.5% Chlorhexidine	Post	30.4 ^*a,b*^	54.6 ^*a,b*^
**Deosan Summer Teat Care**^**^**^	33	0.425% Chlorhexidine	Post	15.6 ^*a,b*^	25.6 ^*b*^
**Deosan Teat Foam Advance**^**^**^	13	0.6% Chlorhexidine	Pre & Post	26 ^*a,b*^	36.5 ^*a,b*^
**Deosan Teatcare Plus**^**^**^	14	0.425% Chlorhexidine	Post	30.3 ^*a,b*^	31. ^*a,b*^
**Hamra Red**^**^**^	12	0.42% Chlorhexidine	Post	20.1 ^*a,b*^	33.5 ^*a,b*^
**Hexa-cel RTU**^**^**^	42	0.52% Chlorhexidine	Pre & Post	61.4 ^*a*^	53.1 ^*a,b*^
**Hexaguard**^**^**^	1	0.74% Chlorhexidine	Pre & Post	22.7 ^*a,b*^	33.6 ^*a,b*^
**Hexaklene R**^**^**^	2	0.5% Chlorhexidine	Pre & Post	58.5 ^*a*^	32.5 ^*a,b*^
**Hexa-Spray**^**^**^	82	0.5% Chlorhexidine	Pre & Post	44.6 ^*a,b*^	49.3 ^*a,b*^
**Kenocidin Spray & Dip**^**^**^	9	0.5% Chlorhexidine	Post	13.1 ^*b*^	33.5 ^*a,b*^
**Kenomint**^**^**^	77	0.5% Chlorhexidine	Post	16.4 ^*a,b*^	24.9 ^*b*^
**Kenomint SD**^**^**^	78	0.5% Chlorhexidine	Post	36.6 ^*a,b*^	31.8 ^*a,b*^
**Masodip Platinum**^**^**^	84	0.436% Chlorhexidine	Pre & Post	30.2 ^*a,b*^	35.3 ^*a,b*^
**PureChem Chlorhexidine**^**^**^	48	1.49% Chlorhexidine	Post	17.0 ^*a,b*^	43.7 ^*a,b*^
**PureChem Chlorhexidine Summer grade**^**^**^	52	1.49% Chlorhexidine	Pre & Post	54.4 ^*a,b*^	50.6 ^*a,b*^
**Quatro**^**^**^	41	0.5% Chlorhexidine	Pre & Post	36.8 ^*a,b*^	31.4 ^*a,b*^
**SensoDip**^**^**^	16	0.5% Chlorhexidine	Pre & Post	16.1 ^*a,b*^	30.9 ^*a,b*^
**Sensodip 50**^**^**^	72	0.5% Chlorhexidine	Post	24.7 ^*a,b*^	18.8 ^*b*^
**Sensospray**^**^**^	66	0.5% Chlorhexidine	Post	45.8 ^*a,b*^	47.1 ^*a,b*^
**Summer C-Dip**^**^**^	58	0.5% Chlorhexidine	Post	42.1 ^*a,b*^	56.9 ^*a,b*^
**Supergold**^**^**^	18	0.5% Chlorhexidine	Pre & Post	42.6 ^*a,b*^	59.6 ^*a,b*^
**Surespray**^**^**^	5	0.5% Chlorhexidine	Pre & Post	24.4 ^*a,b*^	55.6 ^*a,b*^
**Teat Gard C**^**^**^	63	0.5% Chlorhexidine	Pre & Post	42.5 ^*a,b*^	50.1 ^*a,b*^
**Chlorine Dioxide Products**					
Bisept^*^	70	0.05% Chlorine dioxide	Pre & Post	46.7 ^*a*^	38.2 ^*a*^
Kenomix^*^	11	0.0157% Chlorine dioxide	Post	61.3 ^*a*^	51.0 ^*a*^
Kenomix SD^*^	89	0.0157% Chlorine dioxide	Post	57.5 ^*a*^	14.0 ^*a*^
Uddergold^*^	90	0.32% Acidified sodium chlorite	Post	30.3 ^*a*^	18.6 ^*a*^
Valiant^*^	95	0.038% Sodium chloride	Post	35.3 ^*a*^	36.2 ^*a*^
**Diamine Products**					
**Super Cow Teat Foam**^**^**^	26	0.6% Diamine	Pre & Post	72.2	94.7
**Iodine Products**					
**D 4 Iodine**^**+**^	19	0.5% Iodine	Post	10.5 ^*b*^	35.1 ^*a,b*^
**Deosan Super Iodip**^**+**^	34	0.5% Iodine	Post	51.4 ^*a,b*^	39.3 ^*a,b*^
**Ioguard**^**+**^	3	0.5% Iodine	Post	51.8 ^*a,b*^	55.8 ^*a,b*^
**Ioklar Multi**^**^**^	92	0.25% Iodine	Pre & Post	35.7 ^*a,b*^	44.4 ^*a,b*^
**Io-Shield D**^**^**^	91	1.35% Iodine	Post	39.0 ^*a,b*^	13.3 ^*b*^
**Io-Shield Spray**^**^**^	93	0.5% Iodine	Post	12.8 ^*a,b*^	50.1 ^*a,b*^
**Luxdip 50B**^**^**^	69	0.5% Iodine	Post	37.0 ^*a,b*^	55.9 ^*a,b*^
**Masocare Platinum**^**^**^	85	0.54% Iodine	Pre & Post	33.7 ^*a,b*^	34.7 ^*b*^
**Masodine 1:3 Concentrate**^**+**^	83	0.5% Iodine	Pre & Post	73.7 ^*a*^	78.1 ^*a*^
**Maxadine C**^**+**^	8	0.5% Iodine	Post	34.9 ^*a,b*^	38.9 ^*a,b*^
**Maxidine RTU**^**^**^	37	0.5% Iodine	Post	55.3 ^*a,b*^	35.5 ^*a,b*^
**PureChem Iodophor**^**+**^	50	0.5% Iodine	Post	27.7 ^*a,b*^	52.2 ^*a,b*^
**Silkdip**^**+**^	24	0.5% Iodine	Post	28.1 ^*a,b*^	65.3 ^*a,b*^
**Iodine and Lactic Acid Products**					
**Gold Glycodip XL**^**^**^	62	0.5% Iodine & 1% L-(+)-lactic acid	Post	71.0 ^*a*^	75.9 ^*a*^
**Lanodip 4 XL**^**^**^	30	0.5% Iodine & 0.5% L-(+)-lactic acid	Post	55.7 ^*a*^	29.6 ^*a*^
**Lanodip Pre-Post**^**^**^	55	0.29% Iodine & 0.8% L-(+)-lactic acid	Pre & Post	30.1 ^*a,b*^	48.4 ^*a*^
**TriCide**^**^**^	56	0.15% Iodine & 1% L-(+)-lactic acid	Post	34.7 ^*a*^	47.1 ^*a*^
**TriCide Gold**^**^**^	57	0.15% Iodine & 1% L-(+)-lactic acid	Pre & Post	12.0 ^*b*^	53.1 ^*a*^
**Lactic Acid Products**					
**Barri-max**^**^**^	65	2.4% L-(+)-lactic acid	Post	50.7 ^*a,b*^	52.3 ^*a*^
**Blu-gard N Spray**^**^**^	15	3.46% L-(+)-lactic acid	Post	40.2 ^*a,b*^	49.2 ^*a*^
**Dairy Pro Ultra Dip**^**^**^	74	3% L-(+)-lactic acid	Post	24.9 ^*a,b*^	28.0 ^*a*^
**DairyLac SA**^**^**^	76	3% L-(+)-lactic acid	Post	3.2 ^*b*^	28.0 ^*a*^
**Deosan Triathalon**^**^**^	81	1.76% L-(+)-lactic acid	Pre & Post	41.9 ^*a,b*^	35.2 ^*a*^
**Flexigard Spray**^**^**^	94	4% L-(+)-lactic acid	Post	60.9 ^*a,b*^	38.3 ^*a*^
**Kenolac**^**^**^	10	3.6% L-(+)-lactic acid	Post	47.5 ^*a,b*^	42.5 ^*a*^
**Kenolac SD**^**^**^	80	3.6% L-(+)-lactic acid	Post	16.8 ^*a,b*^	17.5 ^*a*^
**Kenopure**^**^**^	79	3.2% L-(+)-lactic acid	Pre	36.8 ^*a,b*^	21.5 ^*a*^
**Lacto-cel**^**^**^	35	2.4% L-(+)-lactic acid	Pre & Post	55.8 ^*a,b*^	59.7 ^*a*^
**Lacto-Mil**^**^**^	96	5% L-(+)-lactic acid	Pre & Post	70.1 ^*a*^	66.9 ^*a*^
**Lactospray**^**^**^	4	2.4% L-(+)-lactic acid	Pre & Post	54.1 ^*a,b*^	59.0 ^*a*^
**Salvodip B**^**^**^	71	2.4% L-(+)-lactic acid	Post	46.4 ^*a,b*^	32.9 ^*a*^
**Salvospray**^**^**^	68	2.4% L-(+)-lactic acid	Pre & Post	37.0 ^*a,b*^	45.5 ^*a*^
**Synofilm**^**^**^	88	8% L-(+)-lactic acid	Post	42.3 ^*a,b*^	36.0 ^*a*^
**Lactic Acid and Chlorhexidine Products**					
**Arkshield**^**^**^	7	5% L-(+)-lactic acid & 0.3% Chlorhexidine	Pre & Post	43.4 ^*a,b*^	64.1 ^*a,b,c,d*^
**Bacto-Lac**^**^**^	31	5% L-(+)-lactic acid & 0.05% Chlorhexidine	Pre & Post	4.3 ^*a,b*^	13.0 ^*d,e*^
**Blue Barrier Spray**^**^**^	49	2% L-(+)-lactic acid & 0.6% Chlorhexidine	Post	54.1 ^*a*^	78.6 ^*a,b*^
**Co-op Source Duo Teat Shield**^**^**^	39	2% L-(+)-lactic acid & 0.3% Chlorhexidine	Pre & Post	0.4 ^*b*^	60.7 ^*a,b,c,d*^
**Dual Dip**^**^**^	45	2% L-(+)-lactic acid & 0.3% Chlorhexidine	Pre & Post	30.0 ^*a,b*^	56.3 ^*b,c,d,e*^
**Dual Dip Supreme**^**^**^	47	2% L-(+)-lactic acid & 0.6% Chlorhexidine	Pre & Post	50.7 ^*a,b*^	100 ^*a*^
**Duo-cel**^**^**^	38	2.5% L-(+)-lactic acid & 0.3% Chlorhexidine	Pre & Post	46.6 ^*a,b*^	64.5 ^*a,b,c,d*^
**Duogold**^**^**^	17	2% L-(+)-lactic acid & 0.3% Chlorhexidine	Pre & Post	18.6 ^*a,b*^	41.8 ^*b,c,d,e*^
**Duo-Teat Shield**^**^**^	25	2% L-(+)-lactic acid & 0.3% Chlorhexidine	Pre & Post	10.6 ^*a,b*^	11.4 ^*d,e*^
**Fortress Protect Film**^**^**^	73	3% L-(+)-lactic acid & 0.2% Chlorhexidine	Post	18.9 ^*a,b*^	51.6 ^*b,c,d,e*^
**Hypraspray**^**^**^	87	2% L-(+)-lactic acid & 0.03% Chlorhexidine	Pre & Post	0.6 ^*b*^	22.4 ^*c,d,e*^
**Lacto dual**^**^**^	36	2.5% L-(+)-lactic acid & 1.5% Chlorhexidine	Pre & Post	6.0 ^*a,b*^	16.6 ^*d,e*^
**Nano Dual**^**^**^	28	1.93% L-(+)-lactic acid & 0.2% Chlorhexidine	Pre & Post	49.4 ^*a,b*^	52.0 ^*b,c,d,e*^
**Novo Dual**^**^**^	29	4% L-(+)-lactic acid & 0.27% Chlorhexidine	Pre & Post	26.5 ^*a,b*^	27.8 ^*c,d,e*^
**Novodip**^**^**^	60	4.9% L-(+)-lactic acid & 1.28% Chlorhexidine	Post	45.3 ^*a,b*^	7.8 ^*e*^
**Novospray**^**^**^	54	4.9% L-(+)-lactic acid & 0.3% Chlorhexidine	Post	44.6 ^*a,b*^	32.8 ^*b,c,d,e*^
**Protect Pre Post**^**^**^	75	3% L-(+)-lactic acid & 0.25% Chlorhexidine	Pre & Post	46.2 ^*a,b*^	71.8 ^*a,b,c*^
**PureChem Dual Dip**^**^**^	51	1% L-(+)-lactic acid & 1.49% Chlorhexidine	Pre & Post	9.1 ^*a,b*^	62.9 ^*a,b,c,d*^
**Salvohex**^**^**^	67	2% L-(+)-lactic acid & 0.3% Chlorhexidine	Post	32.8 ^*a,b*^	44.9 ^*b,c,d,e*^
**Supreme**^**^**^	64	2.5% L-(+)-lactic acid & 0.375% Chlorhexidine	Pre & Post	28.3 ^*a,b*^	51.3 ^*b,c,d,e*^
**Sure spray Duo**^**^**^	6	2% L-(+)-lactic acid & 0.3% Chlorhexidine	Pre & Post	40.6 ^*a,b*^	81.6 ^*a,b*^
**Lactic Acid and Hydrogen Peroxide Products**					
**Lactic Lather**^**^**^	46	1.6% L-(+)-lactic acid & hydrogen peroxide	Pre	89.9	59.4
**Lactic Acid and Salicylic Acid Products**					
**Biolac PrePost**^**^**^	59	0.25% L-(+)-lactic acid & 0.03% Salicylic acid	Pre & Post	34.6 ^*a,b*^	48.7 ^*a,b*^
**Dermalac Emprasan**^**^**^	27	0.25% L-(+)-lactic acid & Salicylic acid	Pre & Post	4.6 ^*b*^	18.3 ^*b*^
**Emprasan dual**^**^**^	53	0.25% L-(+)-lactic acid & Salicylic acid	Pre & Post	53.9 ^*a,b*^	53.1 ^*a*^
**Hypred Quick Spray**^**^**^	20	2% L-(+)-lactic acid & 0.1% Salicylic acid	Pre & Post	35.0 ^*b*^	37.9 ^*a,b*^
**Lely Quaress-Cura**^**^**^	43	3% L-(+)-lactic acid & Salicylic acid	Post	31.3 ^*a,b*^	33.3 ^*a,b*^
**Prefoam+**^**^**^	21	2% L-(+)-lactic acid & 0.1% Salicylic acid	Pre	25.1 ^*b*^	37.9 ^*a,b*^
**Virolac Concentrate**^**+**^	22	2% L-(+)-lactic acid & 0.1% Salicylic acid	Pre & Post	32.3 ^*a,b*^	35.0 ^*a,b*^
**Virolac Film**^**^**^	23	2% L-(+)-lactic acid & 0.1% Salicylic acid	Post	77.6 ^*a*^	49.6 ^*a*^
**Virolac Spray**^**^**^	86	2% L-(+)-lactic acid & 0.1% Salicylic acid	Pre & Post	53.1 ^*a.b*^	44.0 ^*a,b*^

^^^RTU^+^Concentrate ^*^Requires activation before used

^1^ Ingredient/working solution as declared by the manufacturer

^*a −e*^ Inhibition not sharing the same superscript in a column within an ingredient group were significantly different (*P* < 0.05)

Teat disinfection products listed is not an indication of the regulatory status of the products. Check the department of Agriculture, Food and Marine (DAFM) Biocidal Products Register (http://www.pcs.agriculture.gov.ie/registers/biocidalproductregisters/) and Health Products Regulatory Authority (HPRA) (http://www.hpra.ie/homepage/veterinary) before purchase

### Chlorhexidine

In the study, the chlorhexidine ingredient group was the largest, with 25 different products containing various concentrations of chlorhexidine (range: 0.42% w/w to 1.49% w/w chlorhexidine) (Table 1). For streptococcal isolates, product 40 (0.5% w/w chlorhexidine) obtained the largest bacterial reduction of 65.7%, significantly greater than product 9 (13.1%) (0.5% w/w chlorhexidine) (P < 0.05). For staphylococcal isolates, Product 61 (0.53% w/w chlorhexidine) resulted in the greatest reduction of 82.5%, significantly greater than product 72 (18.8%) (0.5% w/w chlorhexidine) (P < 0.05).

### Chlorine Dioxide

Overall, 6 different chlorine dioxide-based products, with concentrations of chlorine dioxide ranging from 0.0157% w/w to 0.32% w/w were tested. No significant differences were observed for both streptococcal and staphylococcal isolates among chlorine dioxide products. For streptococcal isolates, product 11 (0.0157% w/w chlorine dioxide) resulted in the numerically largest bacterial reduction of 61.3% (Table 1). Additionally, product 11 resulted in the numerically greatest bacterial reduction of 51.0% for staphylococcal isolates. Chlorine dioxide products also resulted in reductions comparable to iodine for streptococcal isolates.

### Diamine

Just one product tested contained the ingredient diamine (product 27; 0.6% w/w diamine). This product resulted in a bacterial reduction of 72.2% and 94.7% for streptococcal and staphylococcal isolates, respectively (Table 1).

### Iodine

Thirteen different iodine products of varying concentrations (range: 0.5% w/w to 1.35% w/w iodine) were tested on the teat skin to determine their bacterial reduction. For streptococcal and staphylococcal isolates, product 83 (0.5% w/w iodine) resulted in the largest bacterial reduction of 73.7% and 78.1%, respectively (P < 0.05) (Table 1).

### Iodine and Lactic Acid

In the study, 5 products which contained a combination of iodine and lactic acid were tested. Minor differences in effectiveness were observed within this ingredient group regarding the concentration levels of active ingredients within products. These products ranged from concentrations of 0.15% w/w iodine combined with 0.8% w/w lactic acid to 0.5% w/w iodine combined with 1% w/w lactic acid. Product 62 (0.5% w/w iodine combined with 1% w/w lactic acid) resulted in the largest bacterial reduction of 71.0% in comparison to product 57 (12.0%) (0.15% w/w iodine combined with 1% w/w lactic acid) for streptococcal isolates (*P* < 0.05). Similar to streptococcal isolates, product 62 resulted in the largest bacterial reduction of 75.3% for staphylococcal isolates, which was significantly different to product 30 (29.6%) (0.5% w/w iodine combined with 0.5% w/w lactic acid) (*P* < 0.05). For staphylococcal isolates, the ingredient group, iodine and lactic acid, resulted in bacterial reductions comparable to iodine.

### Lactic Acid

Fifteen products tested contained lactic acid as the main active ingredient. These products ranged from concentrations of 1.76% w/w to 8% w/w lactic acid. No significant differences were observed between lactic acid products for staphylococcal isolates. For streptococcal isolates, product 96 (5% w/w lactic acid) resulted in the numerically largest bacterial reduction of 70.1%.

### Lactic Acid and Chlorhexidine

The second largest ingredient group consisted of 21 products which contained a combination of lactic acid and chlorhexidine. These products ranged in concentrations of 1% w/w lactic acid combined with 0.03% w/w chlorhexidine to 5% w/w lactic acid combined with 1.49% w/w chlorhexidine. Among the lactic acid combined with chlorhexidine products tested, product 49 (lactic acid combined with 0.6% w/w chlorhexidine) resulted in the largest bacterial reduction of 54.1% against streptococcal isolates, which was significantly greater than product 39 (0.4%) (2% w/w lactic acid combined with 0.3% w/w chlorhexidine) (P < 0.05). Product 47 (2% lactic acid combined with 0.6% w/w chlorhexidine) obtained the largest bacterial reduction of 100% against staphylococcal isolates.

### Lactic Acid and Hydrogen Peroxide

One teat disinfection product within the 96 products tested contained a combination of lactic acid and hydrogen peroxide. Product 46 (1.6% w/w lactic acid combined with hydrogen peroxide) was found to result in bacterial reductions of 89.9% and 59.4% for streptococcal and staphylococcal isolates, respectively.

### Lactic Acid and Salicylic Acid

A total of 10 products containing a combination of lactic acid and salicylic acid were tested. These products ranged from concentrations of 0.5% w/w lactic acid combined with 0.03% w/w salicylic acid. For streptococcal isolates, product 23 (2% w/w lactic acid combined with 0.1% w/w salicylic acid) resulted in the numerically largest bacterial reduction of 77.6%. For staphylococcal isolates, product 53 (0.25% w/w lactic acid combined with salicylic acid) resulted in the largest bacterial reduction of 53.1%, which was significantly different to product 27 (18.3%) (0.25% w/w lactic acid combined with salicylic acid) (P < 0.05).

## Discussion

In the current study, streptococcal isolate numbers on teat skin were higher than those observed for staphylococcal isolates. This may be due to the fact that cows were housed indoors during the study. Bedding material can often serve as a point of exposure to environmental organisms, as bedding materials may contain different distributions of microorganisms [32]. Despite the fact that *Staphylococcus aureus* has been identified as the most common subclinical mastitis related pathogen on dairy farms in Ireland [33], a study by Keane et al. [5] found a higher frequency of *Strep uberis* isolated in clinical milk samples collected from 30 dairy herds in Ireland compared to *Staph*. *aureus*.

The day by day variation in naturally present bacterial levels observed on the teat skin would be expected as environmental factors can affect the level of naturally occurring bacterial contamination of the teat skin surface [34, 35]. This may be a limitation of the teat swabbing method as results may vary due to the natural fluctuations in bacterial levels present on the teat skin.

Previous studies have tested pre-milking teat disinfectant products on the teat skin surface by allowing a contact time of 15 s [26] or 30 s [25]. A study by Lopez-Benavides et al. [19], used contact times of 15 s, 30 s and two minutes in a modified version of the BS EN 1656 method found that teat disinfectant products (hydrogen peroxide and chlorine dioxide; recommended for pre- and post-milking disinfection) achieved the required log reduction of greater than 5 against a range of mastitis-causing bacteria within 15 s and 30 s. In the current study, a contact time of one minute was chosen to test both pre- and post-milking teat disinfectant products.

Concentrations of specific ingredients did not always result in the highest level of bacterial reduction as would be expected. While commercially available teat disinfectant products may appear to use similar and/or different concentrations of active ingredients, the levels and strengths of additional ingredients such as emollients may influence the effectiveness of a teat disinfection ingredient [36], while improving teat condition [37]. Limited information concerning emollient levels in products was available; therefore, the impact of those ingredients on teat condition could not be evaluated.

Iodine has been proven to be an effective teat disinfectant against staphylococcal species in both laboratory and field studies [23, 28, 29]. A wide range of new product types and combination of ingredients are now available for teat disinfection. In a previous study, iodine combined with lactic acid and a lactic acid (2.4%) only product achieved a 73% and 79% reduction of naturally present staphylococcal isolates, respectively, on the teat skin, as compared to 76% obtained by an iodine only product [27]. In the current study, the ingredient group iodine combined with lactic acid and lactic acid only achieved reductions which were comparable to the ingredient group, iodine.

Furthermore, iodine has been very effective against streptococcal species in previous studies. A study evaluating the application of iodine to the teat skin found that streptococci species were reduced by 89.77% and no statistical difference was observed in effectiveness between different concentrations [40]. Results of the current study agree with Enger et al. [28] who observed log reductions of 86.4% and 96.2% for *Str*. *uberis* and *Str*. *dysgalactiae*, respectively, when a 0.5% iodophor teat disinfectant product was applied in an excised teat test method. Products containing various levels of lactic acid have been shown to be comparable to iodine against streptococcal isolates in the current study. Previous studies have demonstrated lactic acid to be effective against streptococcal bacteria. A foaming solution containing lactic acid only significantly reduced *Str*. *uberis* on the teat skin with the same product having a reduction of 3.5 times on colonies on the teat skin [12]. Additionally, a 2% lactic acid combined with 0.1% salicylic acid product achieved a cfu/mL reduction of 63% against streptococcal isolates naturally present on the teat skin [27].

In the present study, a chlorhexidine only teat disinfectant product achieved one of the highest log reductions (82.5%) against staphylococcal isolates on the teat skin. Teat disinfectant products containing chlorhexidine have been previously demonstrated to be effective at reducing staphylococcal counts on teat skin [27, 39]. A chlorhexidine product was shown to be 4.46 times more likely to reduce the staphylococcal counts on teat skin in comparison to wash and drying [40]. In a previous study, a 0.5% chlorhexidine resulted in a reduction of 93%, with a product containing 2% lactic acid combined with 0.3% chlorhexidine resulting in a reduction of 71% respectively against naturally present staphylococcal isolates on the teat skin [27].

Within the study, two ingredient groups only included one product each. These ingredient groups were diamine and lactic acid combined with hydrogen peroxide. Authors recognise that this may not allow for an accurate comparison of these ingredient groups against the other seven ingredient groups. However, these individual products resulted in some of the highest bacterial reductions for each bacterial isolate group. Diamine was found to be the most effective against staphylococcal isolates. Furthermore, this product was previously tested using the disc diffusion method and resulted in some of the lowest zones of inhibition against *Staph*. *aureus* (ATCC® 6538™) (Unpublished work, 2020). This ingredient has been known to be stable at a wide range of pH and effective in the presence of heavy organic soiling [41] which may explain why the ingredient was less affected by the organic matter present on teat skin in comparison to other ingredients. Lactic acid combined with hydrogen peroxide was the most effective against streptococcal isolates with a percentage log reduction of 89.9%. A previous study by Enger et al. [28] which used the excised teat method to determine the effect of a 1% hydrogen peroxide (H_2_O_2_) against two streptococcus strains (*Str*. *dysgalactiae* and *Str*. *uberis*) found that this product achieved a high kill effect within 15 s of contact time compared with iodophor products tested. This was also demonstrated by Lopez-Benavides et al. [19] who found that two products, which both contained 0.5% hydrogen peroxide, achieved a > 5 log reduction in a modified BS EN 1656 method. However, at present, hydrogen peroxide has been mainly used in the dairy industry for the cleaning of milking machines and bulk milk tanks. Due to the ingredients history, the prolonged use and concentration of this ingredient within teat disinfectant may have a negative impact on teat skin condition; therefore, the inclusion of the ingredient in a teat disinfectant product must be thoroughly tested to determine its effect on teat skin condition.

The results showed that the ingredient groups; chlorine dioxide, iodine combined with lactic acid and lactic acid only, achieved high bacterial reductions for streptococcal isolates. Whereas, the ingredient groups iodine combined with lactic acid, iodine only and lactic acid combined with chlorhexidine resulted in large bacterial reductions against staphylococcal isolates. However, the individual products containing a combination of lactic acid hydrogen peroxide and lactic acid combined with salicylic acid resulted in the largest bacterial reductions for streptococcal isolates, with products containing a combination of lactic acid and 0.6% w/w chlorhexidine and diamine resulting in the largest bacterial reductions for staphylococcal isolates.

Teat skin condition was not evaluated in this study as products were not applied for a sufficient period of time to have allowed a correct assessment of each individual teat disinfectant products effect on teat skin condition. However, teat skin condition is important and can be regarded as a vital aspect of teat disinfection [38], thus further research must be performed for each individual product used in the study to determine their impact.

## Conclusions

To our knowledge, this is the first reported study to perform an independent analysis of the effectiveness of various teat disinfectant products which are commercially available in Ireland against bacterial isolates naturally present on teat skin in an Irish dairy herd. No statistical difference was observed between the iodine, chlorhexidine or lactic acid groups for both staphylococcal and streptococcal isolates. When products were observed individually, two products (product 46 and product 26) were amongst the most effective for both streptococcal and staphylococcal isolates. This study suggests that some teat disinfectant products achieve a higher reduction in bacterial levels against different specific bacterial groups on teat skin than other teat disinfectant products. Therefore, when choosing a teat disinfectant product, the bacteria in the dairy herds’ environment should be considered. No relationship was observed between a higher concentration of active ingredient and increased effectiveness. Further studies must be performed to evaluate products efficacy against new IMIs and any possible effects on teat skin condition.

## Data Availability

The datasets used and/or analysed during the current study are available from the corresponding author on reasonable request.

## Abbreviations

Conc.: Concentrate
DAFM: Department of Agriculture, Food and Marine
EN: European standard
HPRA: Health Products Regulatory Authority
IMI: Intramammary infection
LF: Left front
LH: Left hind
RF: Right front
RH: Right hind
PBS: Phosphate-buffered saline
PRE: Swab sample collected before teat disinfection
POST: Swab sample collected after teat disinfection
RTU: Ready-to-use
SEM: Standard error mean
TSB: Tryptic soy broth
